# Chemical Profile, Antioxidant, Anti-Proliferative, Anticoagulant and Mutagenic Effects of a Hydroalcoholic Extract of Tuscan *Rosmarinus officinalis*

**DOI:** 10.3390/plants10010097

**Published:** 2021-01-06

**Authors:** Stefania Lamponi, Maria Camilla Baratto, Elisabetta Miraldi, Giulia Baini, Marco Biagi

**Affiliations:** 1 Department of Biotechnologies, Chemistry and Pharmacy, University of Siena, Via Aldo Moro 2, 53100 Siena, Italy; mariacamilla.baratto@unisi.it; 2 Department of Physical Sciences, Earth and Environment, University of Siena, Strada Laterina 8, 53100 Siena, Italy; elisabetta.miraldi@unisi.it (E.M.); baini3@student.unisi.it (G.B.); marco.biagi@unisi.it (M.B.)

**Keywords:** antioxidant activity, antiproliferative activity, mutagenicity, anticoagulant activity, *Rosmarinus officinalis*, hydroalcoholic extract

## Abstract

This study aimed to characterize the chemical profile of an ethanolic extract of Tuscan *Rosmarinus officinalis* (*Ro*ex) and to determine its in vitro bioactivity. The content of phenolic and flavonoid compounds, hydroxycinnamic acids and triterpenoids was determined, and high-performance liquid chromatography-diode array detection (HPLC-DAD) analysis revealed that rosmarinic acid and other hydroxycinnamic derivatives were the main constituents of the extract. *Ro*ex demonstrated to have both antioxidant activity and the capability to scavenge hydrogen peroxide in a concentration dependent manner. Moreover, NIH3T3 mouse fibroblasts and human breast adenocarcinoma cells MDA-MB-231 viability was influenced by the extract with an IC_50_ of 2.4 × 10^−1^ mg/mL and 4.8 × 10^−1^ mg/mL, respectively. The addition of *Ro*ex to the culture medium of both the above cell lines, resulted also in the reduction of cell death after H_2_O_2_ pre-treatment. The Ames test demonstrated that *Ro*ex was not genotoxic towards both TA98 and TA100 strains, with and without S9 metabolic activation. The extract, by inactivating thrombin, showed to also have an anti-coagulating effect at low concentration values. All these biological activities exerted by *Ro*ex are tightly correlated to its phytochemical profile, rich in bioactive compounds.

## 1. Introduction

Plants have been widely used all over the world for their numerous properties throughout the millennia, and today there is a growing interest in the study of classes of compounds obtained from plant species due to their specific activity, allowing their application for many selected purposes in humans [1]. The role of their natural active principles in the human organism is closely related to their chemical structure (functional group types, number and position related to carbon skeleton, substitution in aromatic ring, stereochemistry, side chain length, saturation, etc.) as the type and number of different classes of molecules in plant extracts influence their bioactivity and consequently their use [2]. For example, terpenoids show antimicrobial, antiviral, antibacterial, anticancer, antimalarial, anti-inflammatory effects; phenolics acids have anticarcinogenic and antimutagenic, anti-inflammation and anti-allergic activities; alkaloids have antispasmodic, antimalarial, analgesic and diuretic activities; flavonoids possess antioxidant, anti-inflammatory, antiviral, antibacterial, antifungal, activities, are cardiovascular and hepato-protective; saponins are antitumor, anti-inflammatory, immunostimulant, anti-hypoglycemic, antihepatotoxic and hepatoprotective, anticoagulant, neuroprotective, antioxidant; tannins are antioxidant, anti-carcinogenic, diuretics, hemostatic, anti-mutagenic, metal ion-chelators, antiseptic [2]

*Rosmarinus officinalis* L. is an aromatic, evergreen plant belonging to the family Lamiaceae, native of Mediterranean regions, where it grows wild, but now widely distributed all over the world. The dark green, needle-like leaves of the plant are usually used as spice for flavoring in food cooking, but rosemary is cultivated not only for its aromatic properties but mainly for its antioxidant activity [3,4,5,6]. Rosemary, in the form of extract derived from the leaves, contains several compounds which have been demonstrated to have antioxidant effects [7]. These compounds belong mainly to the classes of phenolic acids, flavonoids, diterpenes and triterpenes [8]. 

Thanks to their chemical composition, rosemary extracts are used in food and in cosmetic industries for preventing food deterioration and protecting skin from free radical damage, respectively [9,10].

Butylated hydroxy anisole (BHA) and butylated hydroxy toluene (BHT) are the commonly used synthetic antioxidants added to foods to preserve the lipid components from quality deterioration, and to cosmetics to inhibit oxidation or reactions promoted by oxygen, peroxides or free radicals. Their synthetic quality, however, will not produce the same health benefits as natural antioxidants [11]. 

*R. officinalis* is also known to be employed in traditional and complementary alternative medicine in many countries, thanks to its broad range of beneficial health properties such as anti-inflammatory [4], anti-proliferative [12], antibacterial [13], antithrombotic [14], anticancer [15,16], hepatoprotective [17], antidiabetic [18], hypocolesterolemic [19] and antihypertensive [20].

It is well known that the antioxidant and biological properties of rosemary extracts are mainly due to their phytochemical composition which includes phenolcarboxylic acids (rosmarinic acid, caffeic acid, vanillic acid, quinic acid, syringic acid) as the major chemical constituents [21]. Other compounds present are: flavonoids [21,22], diterpenes, [21,23], and triterpenes [21]. Considering the co-presence of all the above classes of chemical compounds in rosemary extracts [6,24], their metabolization [25,26,27] and the bioavailability of their metabolites [28], the bioactivity of rosemary cannot be attributed to a single class of compounds but rather to the synergistic contribution of the various bioactive components and their metabolites (that is to its phytocomplex). 

Moreover, agronomical and processing conditions, used in plant cultivation and extraction respectively, may influence the chemical profile of the herbal preparations and, consequently, their pharmacological activities [29,30,31]. For these reasons, chemical analysis should be always performed in order to correlate the type and amount of phytochemicals present with extract bioactivity so as to better select its specific applications.

This study aimed to analyze the chemical profile of an ethanolic extract obtained from an Italian rosemary collected in Tuscany and to determine its in vitro antioxidant activity, both in non-cellular and cellular systems, anti-proliferative effect towards NIH3T3 mouse fibroblasts and human breast adenocarcinoma cell line MDA-MB231, mutagenicity and interference with human blood coagulation factor, in order to correlate chemical profile of the extract with its wide range of in vitro bioactivity and to prevent unwanted effects when used in humans. 

## 2. Results

### 2.1. Chemical Analysis of Rosmarinus Officinalis Extract (Roex)

#### Extraction Yield and Chemical Characterization of Tuscan *R. officinalis* Extract

The solvent utilized for the production of *Ro*ex was a hydro-alcoholic mixture with 60% *v*/*v* ethanol. As reported in Table 1, the amount of dry extract obtained from each gram of dried rosemary subjected to extraction was 112 mg, and 1 mL of suspension contained 24.3 mg of dry material corresponding to a percentage yield of 11.2%. 

In order to quantify total polyphenols and total flavonoids, the extract was analyzed by means of Folin–Ciocalteu colorimetric assay and direct spectrophotometry, respectively. Total sulfuric-vanillin assay-reactive triterpenoids were also quantified. Rosmarinic acid content was evaluated by means of high-performance liquid chromatography-diode array detection (HPLC-DAD) analysis, that was also used in order to identify other phenolic compounds in *Ro*ex.

The quantifications of total phenolic content, expressed as gallic acid equivalents, of total flavonoids expressed as hyperoside, and of total triterpenoids, expressed as β-sitosterol, were reported in Table 1. The amount of each class of compounds was expressed as mg per gram of dry extract (d.e.). 

**Figure 1:**
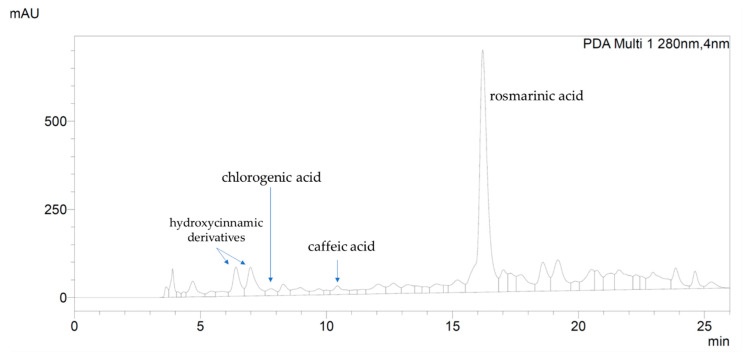
HPLC-DAD analysis of *Ro*ex recorded at 280 nm. Rosmarinic acid is the phenolic compound of the extract with the highest content; other hydroxycinnamic derivatives are present in high concentration.

In order to identify its major components, the extract was analyzed by HPLC-DAD which recorded three main constituents at the following retention time (RT): 6.40 min, 6.95 min and 16.20 min. (Figure 1). By comparing RT and UV spectra of reference standards, it was possible to assign the peak at 16.20 min to rosmarinic acid (Figure 1), while the peak at 6.40 and 6.95 min were assigned to two other hydroxycinnamic derivatives, identified by characteristic UV spectra with λmax at 198–200, 288–298 and 322–332 nm (see Supplementary Materials). Other hydroxycinnamic derivatives identified in *Ro*ex were chlorogenic and caffeic acid, at RT = 7.80 and 10.00 min, respectively. The content of rosmarinic acid, chlorogenic acid, caffeic acid and total hydroxycinnamic derivatives (as the sum of rosmarinic, chlorogenic and caffeic acid and undefined hydroxycinnamic derivatives), expressed as rosmarinic acid, are reported in Table 2. Beside hydroxycinnamic acids, the chromatogram showed two main flavonoids at RT = 18.58 min and 20.55 min, identified by the characteristic UV profile of this class of metabolites with λmax at 194–200, the highest absorbance at 265–290 and a wide shoulder at 330–350 nm (see Supplementary Materials). These flavonoids could not be unambiguously identified, since RT and UV spectrum did not match any used reference standard. Nevertheless, by comparison with published literature on rosemary flavonoids [22,32] and polarity order of identified compounds, the main flavonoids in *Ro*ex could be likely related to luteolin glycosides. 

### 2.2. Antiradical Activity: DPPH Assay and EPR Analysis

The antioxidant behavior of the *Ro*ex was evaluated by its ability to scavenge the free DPPH radical by electron donation, by both spectrophotometric and EPR analysis. The results obtained by spectrophotometric measurements, demonstrated that the extract showed a great antiradical activity with the IC_50_ value around 50 µg/mL.

**Figure 2:**
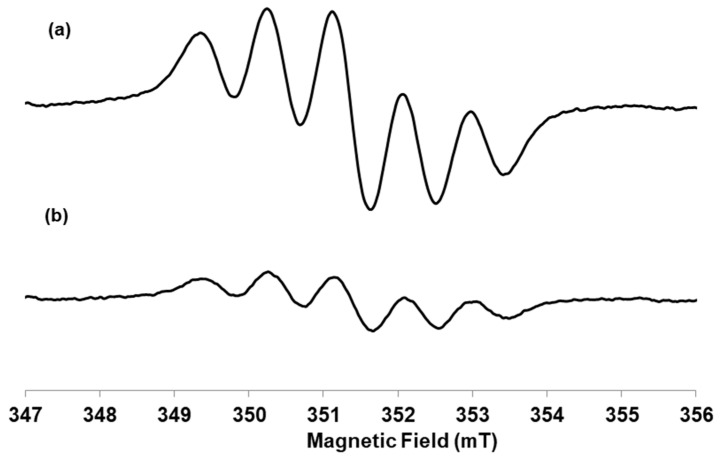
Room temperature X-band spectra of (**a**) DPPH radical alone, (**b**) DPPH radical after the addition of *Rosmarinus officinalis* extract. *Experimental conditions:* The spectra were recorded at microwave frequency ν = 9.86 GHz, microwave power 2 mW and 0.1 mT modulation amplitude.

By EPR analysis, as shown in Figure 2, the addition of the antioxidant to the DPPH radical solution determined a reduction in intensity of the signal with a scavenger percentage equal to 99%. *Ro*ex showed a gallic acid-equivalent antioxidant activity towards DPPH radical equal to 0.777 ± 0.013 (standard deviation).

### 2.3. Hydrogen Peroxide Scavenging Activity

As shown in Table 3, the extract had the capability to scavenge hydrogen peroxide in a concentration dependent manner (0.024–0.96 mg/mL) with an EC_50_ (half maximum effective concentration) of 0.48 mg/mL corresponding at a phenolic content of about 4.6 × 10^−2^ mg/mL, hydroxycinnamic acids content of 2.2 × 10^−2^ mg/mL, flavonoids 3.1 × 10^−3^ mg/mL and triterpenoids 3.4 × 10^−2^ mg/mL. 

### 2.4. In Vitro Anti-Proliferative Activity

**Figure 3:**
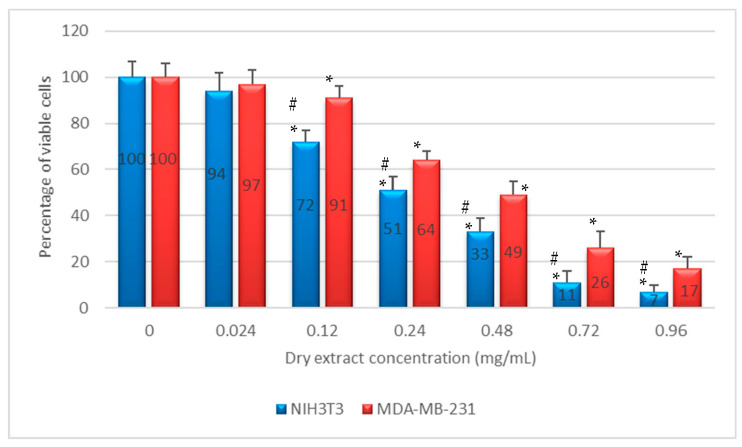
Percentage of viable NIH3T3 and MDA-MB-231 after 24 h of contact with increasing concentration of *Ro*ex dry extracts as determined by the Neutral Red Uptake. Date are mean ± SD of six replicates. * Values are statistically different versus negative control (complete medium), *p* < 0.05. # Values are statistically differently versus percentage of viable MDA-MB-231, *p* < 0.05.

Non-confluent adherent mouse fibroblasts NIH3T3 and human breast adenocarcinoma cells MDA-MB-231 were incubated with different concentrations of *Ro*ex diluted 1:5 with 60% ethanol. Cells were analyzed after 24 h of contact with the test samples and the results are reported in Figure 3. As shown, *Ro*ex exerted an anti-proliferative effect against both NIH3T3 and MDA-MB-231 cell lines in a concentration dependent manner, with lesser efficiency on breast adenocarcinoma cells compared to fibroblasts. 

### 2.5. Protective Effect against Hydrogen Peroxide Induced Oxidative Stress

Concentration-effect relationship for the cytotoxic action of H_2_O_2_ towards NIH3T3 and MDA-MB-231 cells were obtained by testing concentration values of the hydrogen peroxide ranging from 0.082 to 1.60 mM. Cytotoxicity was determined as decreasing of cell viability after H_2_O_2_ pre-treatment followed and not by contact with 7.20 × 10^−3^ mg/mL *Ro*ex, the concentration value at which the extract showed the lowest degree of cytotoxicity for both cell lines.

**Figure 4:**
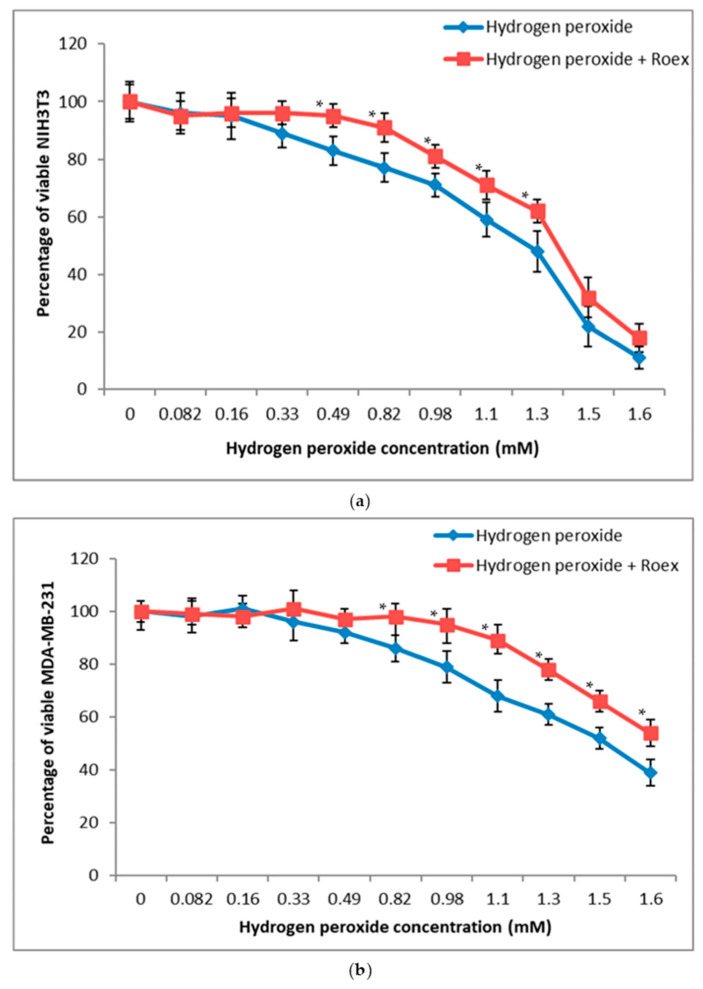
Influence of hydrogen peroxide pre-treatment on viability of (**a**) NIH3T3 and (**b**) MDA-MB-231. * Values are statistically different versus H_2_O_2_ treated cells, *p* < 0.05.

As shown in Figure 4a, *Ro*ex was able to increase significantly NIH3T3 cell viability after pre-treatment with H_2_O_2_ concentrations ranging from 0.33 and 1.3 mM but not at highest values, i.e., 1.5 and 1.6 mM. 

The addition of *Ro*ex increased the percentage of viable MDA-MB-231 after pre-treatment with hydrogen peroxide in a range of concentrations between 0.82 and 1.6 mM (Figure 4b). Human adenocarcinoma breast cells demonstrated to be more resistant to H_2_O_2_ pre-treatment in comparison to NIH3T3 fibroblasts and the rosmarinic extract improved cell viability neutralizing the toxic effect of H_2_O_2_ also at the highest concentrations, demonstrating to be able to protect cells and to reduce cell death. 

### 2.6. Mutagenicity Assay: Ames Test

In *Salmonella* mutagenicity assay, six different concentrations of *Ro*ex were tested by Ames test on TA98, and TA100 strains with and without S9 metabolic activation. The results for the mutagenic effect of *R*oex reported in Table 4 demonstrated that all the concentrations tested were not genotoxic towards both TA98 and TA100 with and without S9 fraction. In fact, also at the highest concentration (24 mg/mL), the number of revertants was lower and statistically different in comparison to positive control (*p <* 0.01). The background level, as well as positive control values, were in all cases within the normal limit found in our laboratory and in accordance with literature data [33]. 

### 2.7. Antithrombotic Activity: Thrombin Time (TT)

By looking at the TT values reported in Table 5, it was possible to note that all the five *Ro*ex tested concentrations significantly increased clotting time in comparison to the control, demonstrating the extract to have anti-coagulating effect also at the lowest concentration value. 

## 3. Discussion

Herbal extracts bioactivity depends on their chemical profile and the choice of the solvent is crucial in extraction processes as it directly affects the chemical composition of the final extracts and the mass extraction yield. Among the alternative solvents available for plants extraction, hydro-alcoholic mixtures are good candidates since they are rather few selective and let to extract a wide range of compounds [34,35,36,37]. Considering global extraction yields of *Rosmarinus officinalis*, a ratio ranging from 50% to 80% ethanol gives highest mass extraction yields and total content in target compounds in comparison to lower Et-OH values. For this reason, the solvent that we utilized for the production of *Ro*ex was hydro-alcoholic mixture with 60% ethanol which yielded 112 mg of dry extract from each gram of rosemary subjected to extraction, corresponding to a percentage yield of 11.2%. This lower percentage yield obtained in comparison to data reported by Jacotet-Navarro et al. [38] can be probably due to the environmental conditions as *Rosmarinus officinalis* is a sensitive plant to pedoclimatic variations that affect its chemical composition [30].

The *Ro*ex, showed a high total phenolic content, of about 95.8 mg/g dry extract, equivalent to 8.55 mg/g dry herbal material ca., in accordance with other extracts obtained from Mediterranean rosemary [21]. As previously reported for other rosemary [22,39], also in *Ro*ex the main phenolic subclass was identified in hydroxycinnamic acid derivatives, and rosmarinic acid was the main single compound, counting almost the half of total phenolic content. Still in accordance with other published papers, also in *Ro*ex total flavonoid content was low, about 6.5 mg/g. This analysis of the phenolic composition of the Italian rosemary extract, performed by means of HPLC-DAD technique, represents a good characterization of its phenolic fingerprint and demonstrated the range of molecules contributing to the definition of this matrix, and may assist in the study of its in vitro bioactive properties [8]. Surprisingly, we found also a high content in triterpenoids in *Ro*ex, 71.7 mg/g extract. Factors such as plant age, climate and stress conditions that inhibit or enhance the production of certain compounds might affect the chemical composition of the extracts [40]. These factors influence the metabolites qualitatively and quantitatively and may explain why certain compounds such as quercetin, rutin and other quercetin glycosides were not detectable in this study [40]. 

The good in vitro antioxidant activity in cell free assays showed by *Ro*ex, demonstrated both by its ability to scavenge the free DPPH radical via electron donation and by the capability to scavenge hydrogen peroxide in a concentration dependent manner (Figure 2), is very similar to others typical vegetal extracts with a good antiradical power [41], and is a consequence of its phytocompounds composition. Hydroxycinnamic acids, presents in high concentration in *Ro*ex, are potent antioxidants and, among them, rosmarinic acid and caffeic acid have a good dose-dependent DPPH scavenging activity and may contribute to the antiradical activity of the extract [42], together with rosmarinic acid. Moreover, also flavonoids and their metabolites are able to scavenge radicals and participate in antioxidant reactions as well as triterpenoids and total phenolic compounds [42]. 

A number of studies have demonstrated a good correlation of intrinsic antioxidant activity in a non-cellular assay with cytoprotection against an oxidant challenge in a cellular assay, demonstrating the ability of antioxidants to act intracellularly [43,44]. Cellular protection of *Ro*ex towards H_2_O_2_ pre-treatment, was evaluated against both cancer and non-cancer cell line, adenocarcinoma breast cancer cells MDA-MB-231 and mouse fibroblasts NIH3T3, respectively (Figure 4). According to data reported in literature [45], *Ro*ex was able to increase significantly NIH3T3 cell viability after pre-treatment with H_2_O_2_ concentrations ranging from 0.33 and 1.3 mM but not at highest values, i.e., 1.5 and 1.6 mM (Figure 4a). Human adenocarcinoma breast cells demonstrated to be more resistant to H_2_O_2_ pre-treatment in comparison to NIH3T3 fibroblasts and the rosmarinic extract improved cell viability neutralizing the toxic effect of H_2_O_2_ also at the highest concentrations (from 0.82 to 1.6 mM), demonstrating to be able to protect cells and to reduce cell death (Figure 4b). The importance of rosmarinic and hydrocynnamic derivative to protect cells in H_2_O_2_-induced cytotoxicity is well known. Rosmarinic acid exhibited substantial H_2_O_2_ scavenging activity and inhibited H_2_O_2_-induced intracellular ROS production [44]. Caffeic acid was found to scavenge intracellular reactive oxygen species, and 1,1-diphenyl-2-picrylhydrazyl radical, and thus prevented lipid peroxidation. Chlorogenic acid protect cells against H_2_O_2_-induced oxidative stress and apoptosis [45]. Intracellular dose-dependent uptake of flavonoids has been demonstrated in various cell types in vitro and is believed to be even higher in vivo than under normal culture conditions [16]. 

In vitro antiproliferative activity of *Ro*ex towards NIH3T3 and MDA-MB-231, noncancer and cancer cells, respectively, was evaluated by NRU test. The NRU results showed that *Ro*ex exerted a weak anti-proliferative effect against both NIH3T3 and MDA-MB-231 cell lines in a concentration dependent manner, with lesser efficiency on breast adenocarcinoma cells compared to fibroblasts (Figure 3). In particular, the concentration value of 2.4 × 10^−1^ mg/mL reduced NIH3T3 viability by 50%, while the same effect with MDA-MB-231 was obtained at a *Ro*ex concentration of 4.8 × 10^−1^ mg/mL, a higher value in comparison to data reported in literature where MDA-MB-231 cells proliferation is inhibited in a dose-dependent manner with an IC_50_ of about 2.04 × 10^−2^ mg/mL [46]. The antiproliferative effect of rosemary extracts towards cancer cells is influenced by their constituents and seems to be correlated with the presence of polyphenols, mainly caffeic and rosmarinic acid [47]. On the contrary, rosmarinic and caffeic acid demonstrated low cytotoxicity against non-cancer cells [48] and this could explain the weak antiproliferative activity of rosemary extract against non-cancer NIH3T3 cells.

A genotoxicity study is a key step for risk assessment during development of natural plant extracts for human applications because various genotoxic compounds can cause a DNA damage compromising human health [49].

In the bacterial reverse mutation assay (Ames test) performed on *Ro*ex, as expected, the positive control agents significantly induced genotoxicity but no genotoxic positive result was observed in *Ro*ex treated groups compared to control at any of the tested concentrations (Table 4). These results indicate that *Ro*ex and their phytochemical components, did not exhibit any genotoxic risk under the experimental conditions of this study. The pre-clinical evaluation of the antithrombotic potential of novel molecules requires the use of reliable reproducible experimental models. TT is one of the most commonly used tests to determine the efficacy of novel antithrombotic drugs. In our experiment, TT using pooled plasma from human healthy patients, was utilized to evaluate the anticoagulant effect of *Ro*ex. The results of TT assay (Table 5) showed that *Ro*ex prolonged coagulation times compared with the control sample, suggesting that the extract inhibited the activity of thrombin.

There is an evidence that coagulation and inflammation are related processes that may considerably affect each other [50]. On the basis of our current experimental studies, it can be hypothesized that inhibitory modulation of coagulation by extracts could give promising anti-inflammatory mediators. These anti-inflammatory and anticoagulant effects have been mostly attributed to the polyphenol and flavonoid compounds found in large quantities in these plants [51] as well as in our *Ro*ex. In the future, well-designed prospective studies are needed to prove this hypothesis.

## 4. Materials and Methods

### 4.1. Materials

Thrombin, Dulbecco’s Modified Eagle’s Medium (DMEM), trypsin solution, and all the solvents used for cell culture as well as Folin–Ciocalteu reactive, DPPH (2,2′-diphenyl-1-picrylhydrazyl), gallic acid and all the other reagents were of analytical grade and purchased from Sigma-Aldrich (Milan, Italy). Mouse immortalized fibroblasts NIH3T3 and human breast adenocarcinoma cells MDA-MB-231 were from American Type Culture Collection (Manassaa, VA, USA). Ames test kit was supplied from Xenometrix (Allschwil, Switzerland). 

### 4.2. Rosmarinus officinalis Extract (Roex) Preparation

*Rosmarinus officinalis* L. (Tuscan blue cultivar, Linnean Herbarium number LINN 41.1, authenticated at the Botanical Museum and Garden of University of Siena) fresh leaves were collected in spring (April) in Val d’Orcia (Tuscany, Italy, 42°56′02″ N 11°38′17″ E). The fresh leaves were washed three times in distilled water and left to dry at room temperature for 24 h. Then, the leaves were chopped with a scalpel. The extract was obtained by putting 20 g of chopped leaves in 80 g of 60% (*v*/*v*) ethanol (EtOH) for 48 h at room temperature in a shaker incubator. At the end of the incubation, the suspension was filtered by a 0.45 µm Whatman membrane filter and dried using a rotary evaporator. The dry extract obtained was weighted and the percentage yield was expressed as air-dried weight of plant material. Samples then were stored at 2–4 °C until it was time to conduct further analysis.

### 4.3. Chemical Analysis

#### 4.3.1. Determination of Total Phenolic and Flavonoid Content

Total polyphenols and flavonoids content of *Rosmarinus officinalis* ethanolic extract (*Ro*ex) was examined using spectrophotometric methods reported by Biagi et al., 2014 [52]. In detail, total polyphenols were determined by the colorimetric method of Folin-Ciocalteu. 0.01 mL of each extract were added to 2.990 mL of distilled water and 0.500 mL of Folin-Ciocalteu reagent 1:10 *v*/*v* in distilled water. After 30 s of shaking, 1.000 mL of Na_2_CO_3_ 15% *m*/*m* in distilled water was added. After incubation at room temperature for 120 min, absorbance at 700 nm was read using a SAFAS UV-MC2 instrument (SAFAS, Monaco, Principality of Monaco). The polyphenols quantification was calculated by means of interpolation of calibration curve constructed using gallic acid. 

Total flavonoids content of extracts was determined reading absorbance at 353 nm of 100 folds diluted extract according with Sosa et al. [53] and constructing calibration curve using hyperoside as standard.

#### 4.3.2. Determination of Total Triterpenes

A total of 10 µL of the sample solution was added to 190 µL of glacial acetic acid, after which 300 µL of a solution of glacial acetic acid with 5% *m*/*v* vanillin and, after mixing for 30 s, 1 mL of perchloric acid.

The mixture was heated to 60 °C for 45 min and, after cooling, the volume was brought to 5 mL with glacial acetic acid [54].

The absorbance was read at 548 nm and the quantification of the total triterpenes in the extract calculated according to the calibration curve, constructed using β-sitosterol.

#### 4.3.3. High-Performance Liquid Chromatography Analysis of Phenolics Compounds

With the aim of further investigating the polyphenolic fraction of the extract, a high-performance liquid chromatography-diode array detection (HPLC-DAD) analysis was carried out. 

A Shimadzu Prominence LC 2030 3D instrument equipped with a Bondapak^®^ C18 column, 10 µm, 125 Å, 3.9 mm × 300 mm column (Waters Corporation, Milford, MA, USA) was used.

Water + 0.1% *v*/*v* formic acid (A) and acetonitrile + 0.1% *v*/*v* formic acid (B) were used as mobile phase. The following program was applied: B from 10% at 0 min to 25% at 20 min, then B 50% at 26 min.; flow was set at 0.8 mL/min. Chromatograms were recorded at 280 nm.

Analyses were performed using 10 µL of extract; rosmarinic acid, caffeic acid, chlorogenic acid, quercetin, apigenin, luteolin, rutin and hyperoside were used as external standard. Calibration curves were established using reference standards ranging from 0.008 mg/mL to 0.500 mg/mL. The correlation coefficient (R^2^) of each curve was > 0.99. 

### 4.4. Antiradical Capacity: DPPH Assay

Antiradical capacity of *Ro*ex was evaluated both by spectrophotometric method using ascorbic acid as reference, and by EPR analysis using gallic acid as reference.

#### 4.4.1. Spectrophotometric Assay

The antiradical capacity of *Ro*ex was tested by means of the validated DPPH (2,2-diphenyl-1-picrylhydrazyl) test. The DPPH solution was prepared in methanol at a concentration of 1 × 10^−4^ M. *Ro*ex was tested in seven 1:2 serial dilutions (ethanol 60% *v*/*v*) and ascorbic acid, used as reference.

All the samples were mixed with the DPPH solution (1:19), transferred into 1 cm path length cuvettes and incubated for 30 min at room temperature in the dark. Ethanol 60% *v*/*v* and DPPH (1:19) was used as positive control. The inhibition of DPPH was calculated according to the following Formula (1) where Absc is the absorbance of the positive control and Absx the absorbance of tested samples IC_50_ was calculated by constructing the curve of inhibition values for each tested concentration (in the linear range 10–75%) [55].

% inibition = (Absc − Absx)/Absc × 100(1)

#### 4.4.2. Scavenger Activity by Electron Paramagnetic Resonance (EPR) Analysis

Continuous-wave X-band (CW, 9 GHz) EPR spectra were recorded using a Bruker E580 ELEXSYS Series spectrometer (Bruker, Rheinstetten, Germany), with the ER4122SHQE cavity. EPR measurements were performed filling 1.0 mm ID × 1.2 mm OD quartz capillaries placing them into a 3.0 mm ID × 4.0 mm OD suprasil tube. 

A stock solution of DPPH was prepared (0.2 mM in ethanol) and the final concentration of the radical in each sample was 0.16 mM. The extract was diluted a hundred times in respect to the stock solution and it was added with one fourth volume in respect to that of the radical. The addition was incubated for 15 min.

The area of the EPR spectra was calculated by the double integral of the DPPH signal and the scavenger percentage was calculated using the Formula (2) where A_0_ is the area of the DPPH signal without the addition of the extract, A_extract_ is the area the DPPH signal after the addition of the extract.

The addition of the antioxidant to the DPPH radical solution determines a reduction in intensity of the EPR signal with a scavenger percentage equal to 99%.

scavenger % = (A_0_ − A_extract_)/A_0_ × 100 (2)

#### 4.4.3. Gallic Acid-Equivalent Antioxidant Activity through DPPH Assay

A stock solution of DPPH radical 1 mM in EtOH was freshly prepared and used within 5 h. A stock solution of gallic acid 0.2 mM in EtOH was prepared. The calibration curves were built using a standard solution of gallic acid. The gallic acid standard solution with different linear increasing volumes (10–100 µL) and a known volume (100 μL) of the extract was added to a fixed volume of the DPPH solution (100 µL). After 15 min of incubation in the dark at room temperature, the EPR spectra were recorded. The antioxidant activity was plotted through the decay percentage of the area of the DPPH signal versus increasing concentrations of gallic acid standard solution. The area of the EPR spectra was calculated through the double integral of the DPPH signal. 

The decay percentage for the plotting refers to the Formula (3) where A_0_ is the area of the DPPH signal without the addition of the antioxidant or extract, A_S_ is the area the DPPH signal after the addition of scavenger agent such as antioxidant gallic acid or the extract.

The decay area percentage expressed in gallic acid equivalent was obtained through the calibration curve built with the standard solution (R^2^ = 0.928) and reporting the area of the DPPH EPR signal after the addition of the extract. The extract was diluted a hundred times in respect to the stock solution. The measurements were repeated in triplicate and the antioxidant activity of the extract was expressed as mole/g of acid gallic equivalent.

decay% = (A_0_ − A_S_)/A_0_ × 100 (3)

### 4.5. Hydrogen Peroxide Scavenging Assay

The ability of *Ro*ex to scavenge hydrogen peroxide was estimated according the method of Ruch et al. [56]. A solution of H_2_O_2_ (2 mM) was prepared in phosphate buffer (50 mM, pH = 7.4). Aliquots (0.05, 0.1, 0.2, 0.3 and 0.4 mL) of *Ro*ex at a concentration of 24 mg/mL, were transferred into test tubes and their volumes were made up to 0.4 mL with 50 mM phosphate buffer at pH = 7.4. After adding 0.6 mL of hydrogen peroxide solution, tubes were vortexed and the absorbance of H_2_O_2_ at 230 nm was determined after 10 min of incubation, against a blank containing phosphate buffer and Et-OH 60% without H_2_O_2_. The percentage of hydrogen peroxide scavenging was calculated as follows:

% scavenged H_2_O_2_ = [(A_i_ − A_t_)/A_i_] × 100 (4)

where A_i_ is the absorbance of control and A_t_ is the absorbance of test samples.

### 4.6. Anti-Proliferative Assay and Protective Effect against Hydrogen Peroxide Induced Oxidative Stress

#### 4.6.1. Cell Cultures and Anti-Proliferative Test

In order to evaluate the in vitro anti-proliferative activity of new products, the direct contact test was used [57]. This test is suitable for sample with various shapes, sizes or physical status (i.e., liquid or solid). The evaluation of in vitro inhibition of cell growth does not depend on the final use for which the product is intended, and the document ISO 10995-5:2009 recommends many cell lines from American Type Collection. Among them, to test *Ro*ex cytotoxicity, NIH3T3 mouse fibroblasts were chosen [58]. Moreover, in order to evaluate the anti-proliferative activity of *Ro*ex towards tumoral cells, the same test was repeated by using human breast adenocarcinoma cells MDA-MB 231.

Both NIH3T3 and MDA-MB231 cells were propagated in DMEM supplemented with 10% fetal calf serum, 1% L-glutamine-penicillin-streptomycin solution, and 1% MEM non-essential amino acid solution, and incubated at 37 °C in a humidified atmosphere containing 5% CO_2_. Once at confluence, the cells were washed with PBS 0.1M, separated with trypsin-EDTA solution and centrifuged at 1000 r.p.m. for 5 min. The pellet was re-suspended in complete medium (dilution 1:15). Cells (1.5 × 10^4^) suspended in 1 mL of complete medium were seeded in each well of a 24 well round multidish and incubated at 37 °C in an atmosphere of 5% CO_2_. Once reached the 50% of confluence (i.e., after 24 h of culture), the culture medium was discharged and the test compounds, properly diluted in completed medium, were added to each well. All samples were set up in six replicates. Complete medium was used as negative control. After 24 h of incubation, cell viability was evaluated by neutral red uptake (NRU) assay [59].

#### 4.6.2. Protective Effect against Hydrogen Peroxide Induced Oxidative Stress

To determine the protective effect of alcoholic extract of *Ro*ex against oxidative stress, NIH3T3 and MDA-MB-231 cells were pre-incubated with different concentrations of hydrogen peroxide (0.1, 0.2, 0.3, 0.9, 1.0, 1.1, 1.3, 1.5, 1.6 µM) for 15 min, then washed and incubated for 24 h with 0.3% *v*/*v Ro*ex at a concentration of 7.2 × 10^−3^ mg/mL in complete culture medium. Cell viability was evaluated after 24 h of incubation at 37 °C in 5% CO_2_ by NRU assay.

#### 4.6.3. Evaluation of Cell Viability: NRU Assay

In order to determine the percentage of viable cells as follows, the following solutions were prepared:

1. Neutral Red (NR) stock solution: 0.33 g NR dye powder in 100 mL sterile H_2_O
2. NR medium: 1.0 mL NR stock solution + 99.0 routine culture medium pre-warmed to 37 °C
3. NR desorb solution: 1% glacial acetic acid solution + 50% ethanol + 49% H_2_O

At the end of incubation, the routine culture medium was removed from each plate and the cells were carefully rinsed with 1 mL pre-warmed D-PBS 0.1M. Plates were then gently blotted with paper towels. 1.0 mL NR medium was added to each dish and further incubated at 37 °C, 95% humidity, 5.0% CO_2_ for 3 h. The cells were checked during incubation for NR crystal formation. After incubation, the NR medium was removed and the cells were carefully rinsed with 1 mL pre-warmed D-PBS 0.1M. PBS was decanted and blotted from the dishes and exactly 1 mL NR desorb solution was added to each sample. Plates were placed on a shaker for 20–45 min to extract NR from the cells and form a homogeneous solution. During this step the samples were covered to protect them from light. Five minutes after removal from the shaker, absorbance was read at 540 nm with a UV/visible spectrophotometer (Varian Cary 1E).

### 4.7. Mutagenicity Assay: Ames Test

The TA100 and TA98 strains of *Salmonella typhimurium* were utilized for mutagenicity assay in absence and presence of metabolic activation, i.e., with and without S9 liver fraction. The tester strains used were selected because they are sensitive and detect a large proportion of known bacterial mutagens and are most commonly used routinely within the pharmaceutical industry [60]. The following specific positive controls were used, respectively, with and without S9 fraction: 2-nitrofluorene (2-NF) 2 µg/mL + 4-nitroquinoline N-oxide (4-NQO) 0.1 µg/mL, and 2-aminoanthracene (2-AA) 5 µg/mL. The final concentration of S9 in the culture was 4.5%.

Approximately 10^7^ bacteria were exposed to 6 concentrations of *Ro*ex, as well as to positive and negative controls, for 90 min in medium containing sufficient histidine to support approximately two cell divisions. After 90 min, the exposure cultures were diluted in pH indicator medium lacking histidine, and aliquoted into 48 wells of a 384-well plate. Within two days, cells which had undergone the reversion to His grew into colonies. Metabolism by the bacterial colonies reduced the pH of the medium, changing the color of that well. This color change can be detected visually. The number of wells containing revertant colonies were counted for each dose and compared to a zero-dose control. Each dose was tested in six replicates.

The material was regarded mutagenic if the number of histidine revertant colonies was twice or more than the spontaneous revertant colonies.

### 4.8. Antithrombotic Activity: Thrombin Time (TT)

The selected blood donors were normal, healthy men who had fasted for more than 8 h and had not received medication for at least 14 days. Blood samples were collected into 3.8% (m/V) tri-sodium citrate as anticoagulant at a volume ratio of 9 parts blood to 1-part citrate. The blood samples were then centrifuged at 3500 r.p.m. for 15 min to obtain PPP which was utilized to perform TT. In particular, 0.2 mL of each samples were added to 0.2 mL of a solution obtained by diluting 1:1 human plasma with a solution of 60% Et-OH (% *v*/*v*). TT was determined by incubating the aliquot (0.2 mL) of human plasma containing the sample at 37 °C for 2 min, after which 0.2 mL of thrombin (0.6 NIH) was added. The clotting time was revealed by an Automatic ElviDigiclot 2 Coagulimeter (from Logos SpA, Milan, Italy).

### 4.9. Statistical Analysis

All assays were carried out in six replicates and their results were expressed as mean ± standard deviation (SD). Multiple comparisons were performed by one-way ANOVA and individual differences tested by Fisher’s test after the demonstration of significant intergroup differences by ANOVA. Differences with *p <* 0.05 were considered significant.

## 5. Conclusions

Taken together, the data obtained demonstrated that hydro-alcoholic extraction of Tuscan rosemary can be a good method for obtaining active compounds with high potential for application in many different fields, such as cosmetics, food or pharmaceutical research. The results of this study have demonstrated, indeed, that the *Ro*ex analyzed possessed good antioxidant and radical scavenging activities when tested in cellular and non-cellular assays, as well as a weak, anti-proliferative effects towards both cancer and noncancer cells, absence of genotoxic and ability to prolong thrombin time. All these bioactivities are tightly correlated to the *Ro*ex chemical profile in a dose dependent manner. In order to prevent unwanted effects when used in humans, it is essential that multiple aspects of the bioactivity of each *Rosmarinus officinalis* extract are tested.

## Figures and Tables

**Table 1:** Extraction yield, total phenolic, flavonoids and triterpenoids content. Results are expressed as mean value ± standard deviation (SD) from three different preparations.

Dry Extract for mL of Suspension (mg/mL ± SD)	Extraction Yield (% ± SD)	Total Phenolic Content (mg GAE/g d.e. ± SD)	Total Flavonoids Content (mg Hyperoside/g d.e. ± SD)	Total Triterpenoids Content (mg β-sitosterol/g d.e. ± SD)
24.3 ± 1.2	11.2 ± 0.5	95.8 ± 1.1	6.5 ± 0.7	71.7 ± 7.2

**Table 2:** Content of rosmarinic, chlorogenic and caffeic acid, and total hydroxycinnamic derivatives, expressed as mg of rosmarinic acid/g of d.e.

Compound	mg/g d.e.
Rosmarinic acid	46.3 ± 5.0
Chlorogenic acid	2.0 ± 0.1
Caffeic acid	0.7 ± 0.1
Total hydroxycinnamic derivatives (expressed as rosmarinic acid)	69.4 ± 5.2

**Table 3:** Total phenolic content and percentage of scavenged hydrogen peroxide as a function of increasing concentrations of *Ro*ex.

Concentration of d.e. (mg/mL)	% Scavenged H_2_O_2_ ± SD	Total Phenolic Content (mg/mL)	Total Hydroxycinnamic Acids Content (mg/mL)	Total Flavonoid Content (mg/mL)	Total Triterpenoids (mg/mL)
0.024	15 ± 3	2.3 × 10^−3^	1.1 × 10^−3^	1.6 × 10^−4^	1.7 × 10^−3^
0.12	28 ± 4	1.2 × 10^−2^	5.6 × 10^−3^	7.8 × 10^−4^	8.6 × 10^−3^
0.24	41 ± 4	2.3 × 10^−2^	1.1 × 10^−2^	1.6 × 10^−3^	1.7 × 10^−2^
0.48	53 ± 6	4.6 × 10^−2^	2.2 × 10^−2^	3.1 × 10^−3^	3.4 × 10^−2^
0.72	66 ± 3	6.9 × 10^−2^	3.3 × 10^−2^	4.7 × 10^−3^	5.2 × 10^−2^
0.96	73 ± 5	9.2 × 10^−2^	4.4 × 10^−2^	6.2 × 10^−3^	6.9 × 10^−2^

**Table 4:** Mean number of revertants in *S. typhimurium* strains 98 and 100, exposed to different concentrations of *Ro*ex with and without S9 fraction. The results are reported as the mean of revertants ± SD.

	*Salmonella typhimurium* TA 98		*Salmonella typhimurium* TA 100	
	Revertant (Mean ± SD)		Revertant (Mean ± SD)	
*Ro*ex d.e. Concentration (mg/mL)	−S9	+S9	−S9	+S9
0	1.33 ± 0.52	1.67 ± 1.21	1.33 ± 1.37	1.33 ± 1.21
2.4 × 10^−2^	1.67 ± 1.53	1.33 ± 0.58	1.33 ± 0.58	1.33 ± 1.53
1.2 × 10^−1^	1.67 ± 1.15	2.33 ± 0.58	1.00 ± 0.00	2.00 ± 0.00
2.4 × 10^−1^	2.00 ± 1.73	3.00 ± 1.00	1.33 ± 1.53	2.33 ± 2.08
4.8 × 10^−1^	2.33 ± 1.53	333 ± 1.15	1.67 ± 2.08	1.67 ± 0.58
7.2 × 10^−1^	2.67 ± 1.15	1.33 ± 1.15.	2.67 ± 1.15	2.33 ± 1.53
24	1.33 ± 1.53	2.33 ± 0.58	2.33 ± 1.15	2.33 ± 0.58
Positive Control	31.33 ± 3.06	34.00 ± 2.00	47.33 ± 1.15	47.67 ± 0.58
Baseline	1.85	2.88	2.54	2.70

**Table 5:** Thrombin Time of different concentration of *Ro*ex. Data are mean ± SD of six replicates. (*) Value statistical different versus control (human plasma diluted 1:1 with 60% *v*/*v* Et-OH solution).

	*Ro*ex d.e. Concentration (mg/mL)						
	0	2.4 × 10^−2^	1.2 × 10^−1^	2.4 × 10^−1^	4.8 × 10^−1^	7.2 × 10^−1^	9.6 × 10^−1^
Thrombin Time (second ± SD)	18 ± 2	>120″ (*)	>120″ (*)	>120″ (*)	>120″ (*)	>120″ (*)	>120″ (*)

## Data Availability

The data presented in this study are available in supplementary material.

## Supplementary Materials

The following are available online at https://www.mdpi.com/2223-7747/10/1/97/s1.
