# Supplementary figures and images for: Chemical Profile, Antioxidant, Anti-Proliferative, Anticoagulant and Mutagenic Effects of a Hydroalcoholic Extract of Tuscan Rosmarinus officinalis
Source: Plants (Basel). 2021 Jan 6;10(1):97. doi: 10.3390/plants10010097 (PMC7825123; doi:10.3390/plants10010097)

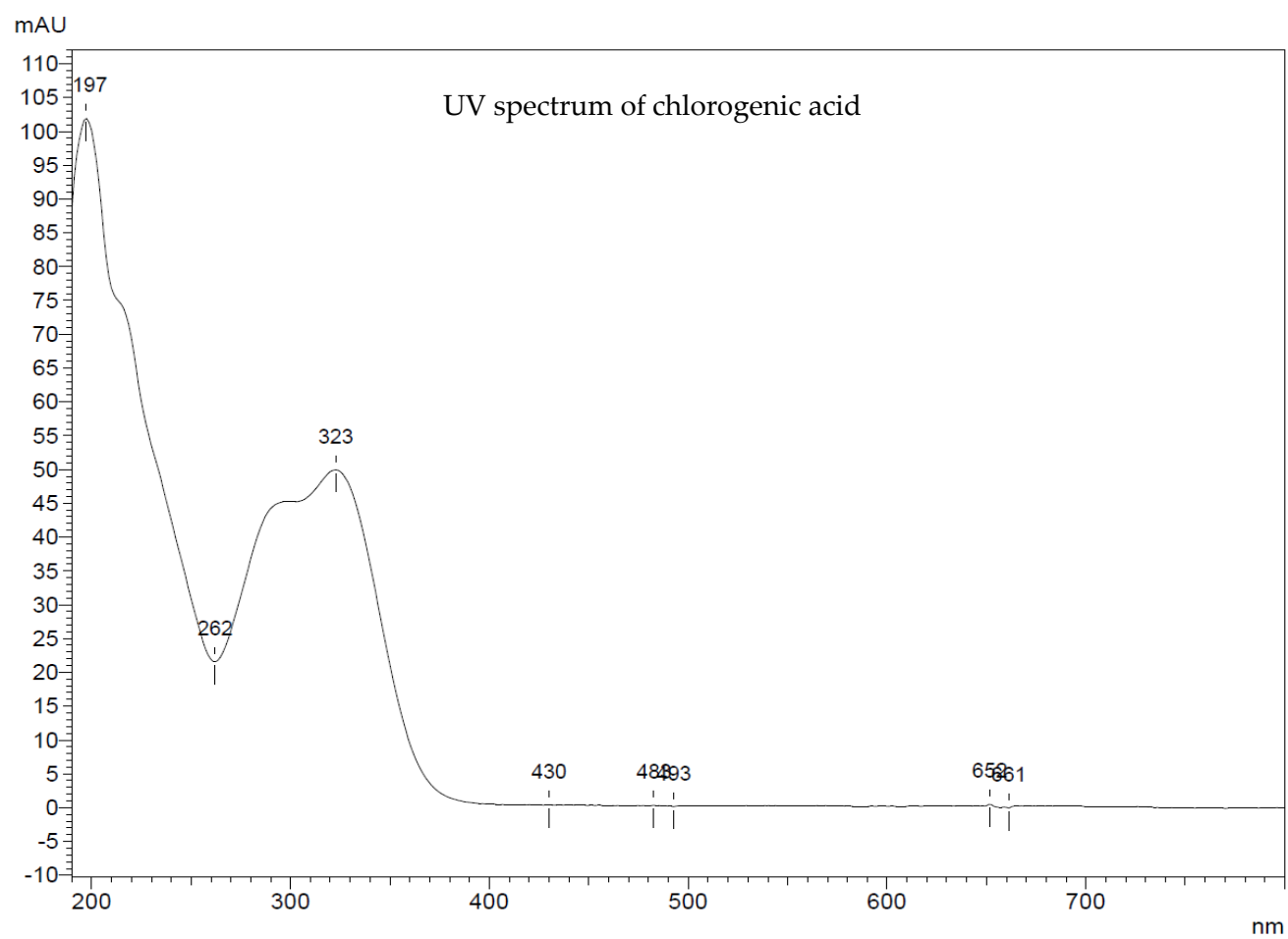

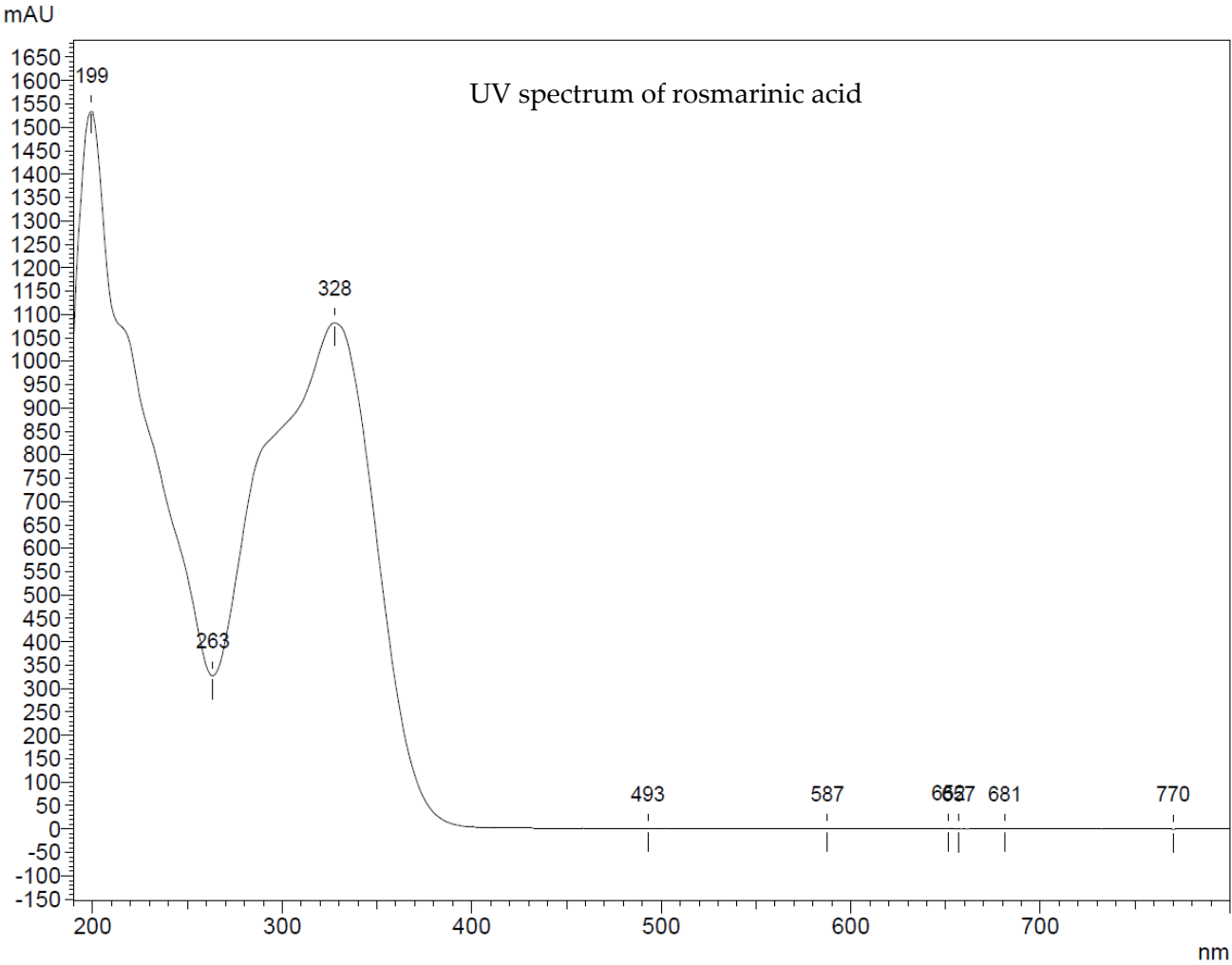

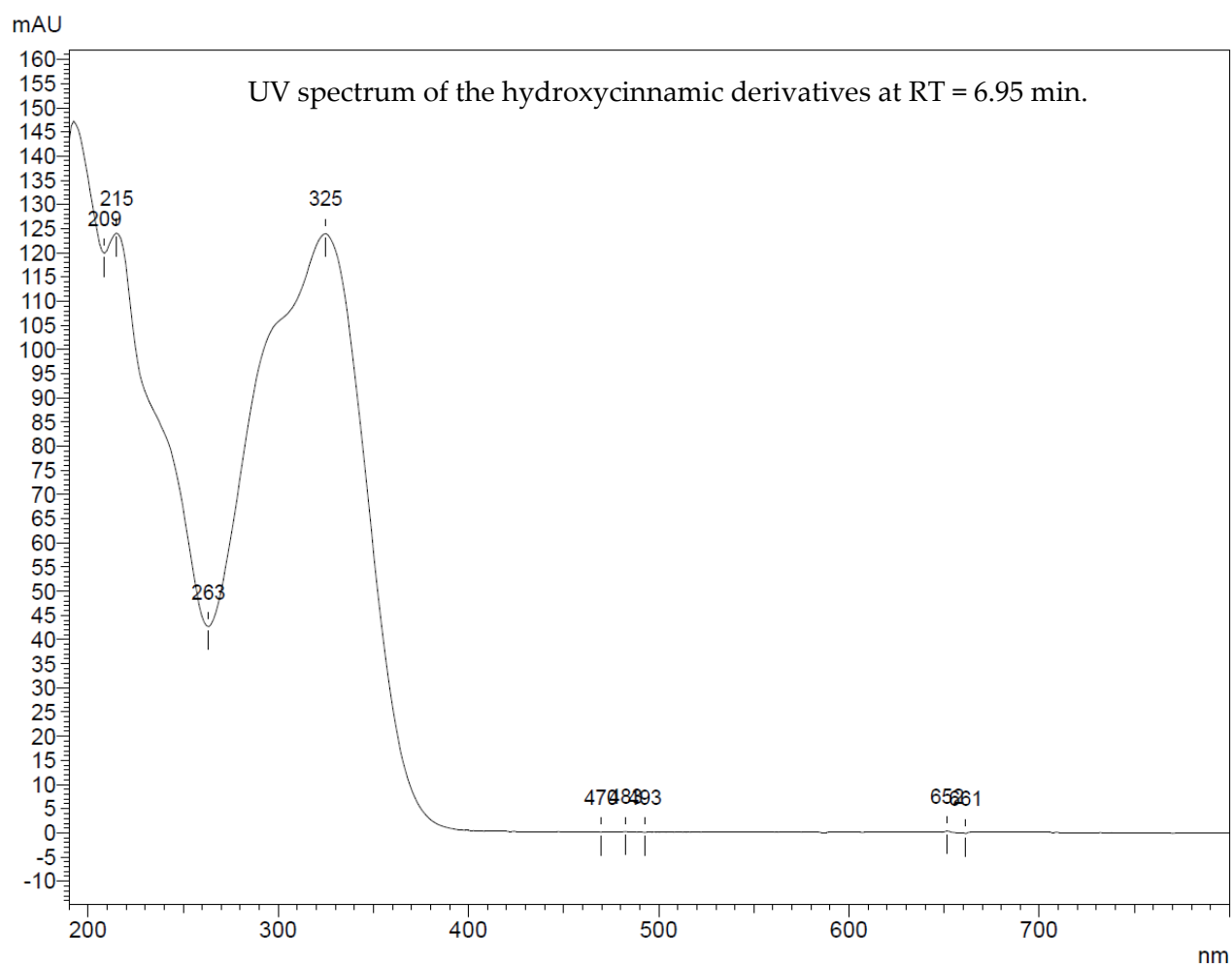

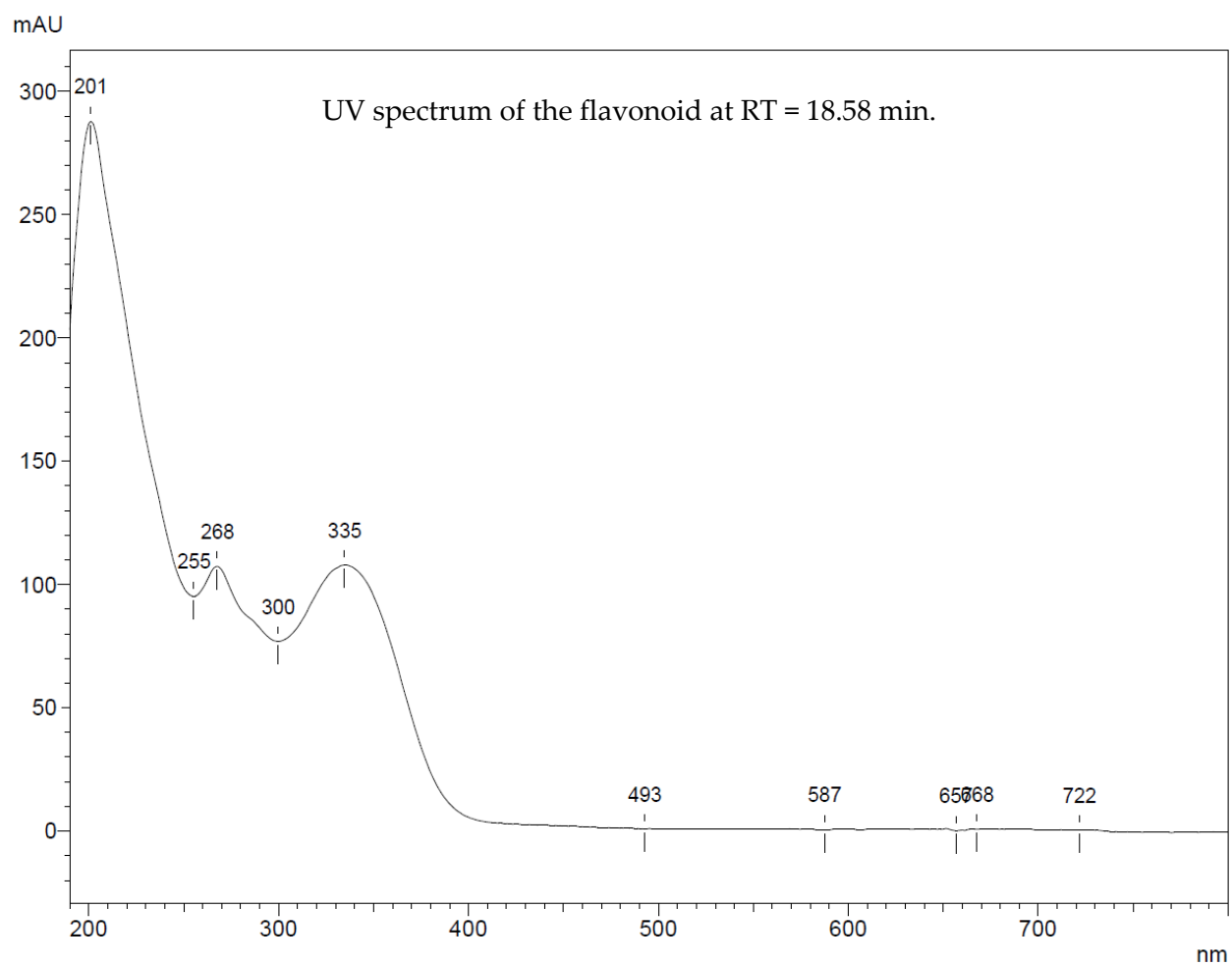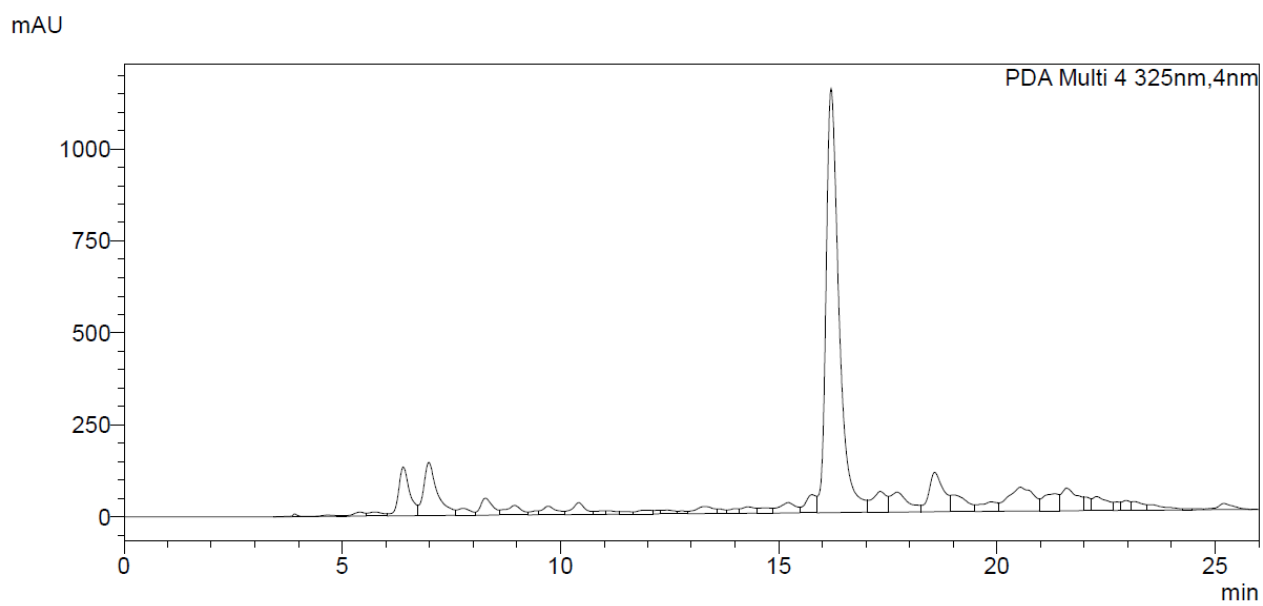

Supplement: Supplementary file 1 [file plants-10-00097-s001.pdf]
